# Supplementary material for: HtrA1 activation is driven by an allosteric mechanism of inter-monomer communication
Source: Sci Rep. 2017 Nov 1;7:14804. doi: 10.1038/s41598-017-14208-z (PMC5666011; doi:10.1038/s41598-017-14208-z)
Supplement: Supplementary file 1 — Supplementary Information [file 41598_2017_14208_MOESM1_ESM.pdf]

HtrA1 activation is driven by an allosteric mechanism of inter-monomer communication

Alvaro Cortes Cabrera[1*], Esther Melo[1*], Doris Roth[1], Andreas Topp[1], Frederic Delobel[1], Corinne Stucki[1], Chia-yi Chen[2], Peter Jakob[1], Balazs Banfai[1,3], Tom Dunkley[1], Oliver Schilling[2,4], Sylwia Huber[1], Roberto Iacone[1], Paula Petrone[1,%]

[1]Pharma Research & Early Development (pRED). Roche Innovation Center Basel, Basel, Switzerland.

[2] Institute of Molecular Medicine and Cell Research, University of Freiburg, 79104 Freiburg, Germany.

[3] Soladis GmbH, 4052 Basel, Switzerland.

[4] BIOSS Centre for Biological Signaling Studies, University of Freiburg, D-79104 Freiburg, Germany

*Authors have contributed equally.

%Paula Petrone. Lead Contact. Address: ppetrone@fpmaragall.org . Barcelonabeta Brain Research Center, Fundacion Pascual Maragall. Carrer de Wellington, 30, 08005 Barcelona, Spain.

Supplemental Information

Index

1. Supplemental Data items: (figures and tables)

1.1. Table S1. Length of molecular dynamics simulations.
1.2. Table S2. Number of dynamic communities obtained depending on the distance threshold used for the monomer simulations.
1.3. Table S3. Number of dynamic communities obtained depending on the distance threshold used for the trimer simulations.
1.4. Table S4. Helicity of the LD loop (residues Pro285, Phe286 and Ser287) in simulation for different variants represented as the percentage of simulation time as part of a helix.
1.5. Table S5. Distribution of the different amino acid types for position Leu345 and Lys346.
1.6. Table S6. Distances from the different loops in the HtrA1 monomer
1.7 Table S7. Description of active monomer communities with a threshold of 6.0 Å. Catalytic residues are highlighted in bold.
1.8. Figure S1. Reaction rate plots for different substrate concentrations for the HtrA1 variants (Wild-type, L345G, K346I, R302A and E306A+R310A).
1.9. Figure S2. Implied timescales plot for the 5-states hidden Markov model.
1.10. Figure S3. Dynamic communities of HtrA1 trimer. Rear view.
1.11. Figure S4. Accessory to Figure 5 in the main text.
1.12. Proteomics methods. Figures S5.
1.13. Supplemental native mass-spectrometry data. Figures S6.
1.14. Figure S7. Results for L3 loop conformation analysis.
1.15. Supplemental Community Analysis HtrA1 monomer
1.16. Supplemental analysis of the L3 loop conformations

2. Supplemental Experimental Procedures and Analyses

2.1. Simulation and modelling details

2.1.1. Simulation

2.1.2. Community analysis

2.1.3. Helicity analysis

2.1.4. Hidden Markov model construction

2.1.5. Residue conservation analysis.

2.2. Statistics for Proteomics

2.3. Western Blot analysis.

3. Supplemental References

4. Supplemental data proteomics: Separate file Supplemental Data Proteomics.xml

5. Supplemental statistical analysis for proteomics: Separate file Supplemental Statistics.

1. Supplemental Data items: (figures and tables)

1.1. Table S1. Length of molecular dynamics simulations. (Related to Supplemental Experimental Procedures 2.1.1)

Starting conformation	Length	Number of independent runs
Wild-type Active	50 ns	2
Wild-type Active	200 ns	4
Wild-type Active	1,000 ns	3
Wild-type Inactive	200 ns	5
Wild-type Inactive	1,000 ns	2
Wild-type Inactive	330 ns	1
Wild-type Active R302A	200 ns	5
Wild-type Active E306A+R310A	200 ns	5
Wild-type Active L345G	200 ns	5
Wild-type Active K346I	200 ns	5
Wild-type Active S284R	200 ns	5
Wild-type Active P285Q	200 ns	5
Wild-type Active F286V	200 ns	5
Total		14.23 µs of trimeric simulations or 42.69 µs for momers.

1.2. Table S2. Number of dynamic communities obtained depending on the distance threshold used for the trimer simulations

	7Å	8Å	9 Å	10 Å	11 Å	12 Å
Active trimer	14	12	11	4	4	4
Inactive trimer	9	11	12	7	3	3
R302A trimer	13	7	6	4	3	5
E306A_R310A trimer	11	9	7	8	6	3
L345G trimer	13	3	3	3	3	3
K346I trimer	15	12	10	4	11	3

1.3. Table S3. Number of dynamic communities obtained depending on the distance threshold used for the monomer simulations.

	5Å	6Å	7Å	8Å	9 Å	10 Å	11 Å	12 Å
Active monomer	8	7	14	12	11	4	4	4

Inactive monomer	9	10	9	11	12	7	3	3

1.4. Table S4. Helicity of the LD loop (residues Pro285, Phe286 and Ser287) in simulation for different variants represented as the percentage of simulation time forming a helix.

Simulation	Percentage of simulation time
Wild-type active conformation	95%
Wild-type inactive conformation	31%
S284R	30%
P285Q	26%
F286V	65%

1.5. Table S5. Distribution of the different amino acid types for position Leu345 and Lys346 with frequency of more than one in the alignment

Leu345 position	Frequency (and %)	Lys346 position	Frequency (and %)
Ala	34 (28.3%)	Lys	51 (42.5 %)
Leu	25 (20.8%)	Ile	37 (30.8%)
Gly	22 (18.3%)	Gly	21 (17.5%)
Met	19 (15.8%)		
Gln	15 (12.5%)		
Ser	2 (1.6%)		

1.6. Table S6. Distances from the different loops in the HtrA1 monomer

Loops	Distance in active comformation (3NZI)	Distance in inactive conformation (3NUM)
L2 – LD	7.40 Å	7.84 Å
L3 – LD	17.27 Å	21.41 Å
L2 – L3	4.46 Å	4.62 Å

1.7 Table S7. Description of active monomer communities with a threshold of 6.0 Å. Catalytic residues are highlighted in bold.

Community	Residues	Number of residues
C1<br><br>(central core, LC loop)	Ile360, Lys359, Asp358, Val221, Thr223, **Asp250**, Lys243, Asp244, Ile242, Glu247, Lys248, Val245, Asp246, Leu253, Ile342, Lys362, Lys361, Leu364, Phe363, Glu366, Thr365, His368, Ser367, Asp369, Gly329, Ala252, Ile251, **Ser328**, **His220**, Ala219, Val216, Asn218, Thr217	33
C2<br><br>(LA loop)	Pro200, Leu188, Arg197, Val199, Phe194, Glu198, Leu192, Lys191, Phe189, Arg190, Val201, Ala202, Lys196, Asn224, Lys225, His226, Arg227, Pro193, Ser195	19
C3	Val222, Val228, Lys229, Val230, Lys241, Lys261, Pro331, His185, Ile186, Glu187, Pro181, Val183, Val184, Phe207, Ile208, Ser205, Gly206, Ser203, Gly204, His258, Asp257, Tyr238, Thr237, Ala236, Gly235,	46

(right core)	Asn234, Lys233, Leu232, Glu231, Pro263, Val264, Ile254, Leu262, Ile256, Lys255, Ala240, Glu239, Gly260, Val209, Gln259, Ile215, Ser210, Asp212, Glu211, Leu214, Gly213	
C4<br><br>(Left core)	Ile296, Ser357, Arg274, Thr294, Thr293, Phe278, Glu277, Glu272, Val297, Ile340, Gly295, Glu338, Gly337, Asp336, Leu335, Asn334, Val333, Ala182, Val280, Leu273, Val279, Val339, Phe171, Ala281, Arg269, Ser270, Leu267, Ala321, Ile322, Leu265, Leu266, Ile172, Gly276, Asp174, Ala173, Val176, Val175, Lys178, Pro275, Ala180, Ile179, Glu177, Val292, Gly268, Ser271	45
C5<br><br>(N-terminal fragment)	Pro162, Ser164, Asn163, Arg166, Leu165, Lys168, His167, Asn170, Tyr169	9
C6<br><br>(L3 loop)	Leu307, Gly308, Lys305, Glu306, Tyr316, Gly304, Arg302, Asn311, Ser312, Met314, Leu309, Arg310, Ala249, Asp313, Gly303, Asp315	16
Com6b<br><br>(LD loop)	Ser284, Ile282, Gly283, Thr291, Pro285, Phe286, Ser287, Leu288, Gln289, Asn290, Asn327, Gly326, Gly330	13
Com7<br><br>(L2 loop, oxyanion hole and peptide-binding site)	Gln318, Ile317, Asp320, Thr319, Ser352, Ile351, Ala354, Phe353, Ser298, Ile355, Thr300, Thr299, Leu332, Thr344, Asn343, Pro356, Gly341, Val347, Thr348, Leu345, Lys346, Ala349, Gly350, Gln301, Tyr325, Ile323, Asn324	27

1.8. Figure S1. Reaction rate plots for different substrate concentrations for the 5 HtrA1 variants: wild-type in black (positive control); L345G in red; K346I in green; E306+R310A in pink; R302A in blue; and S328A (negative control) in orange.

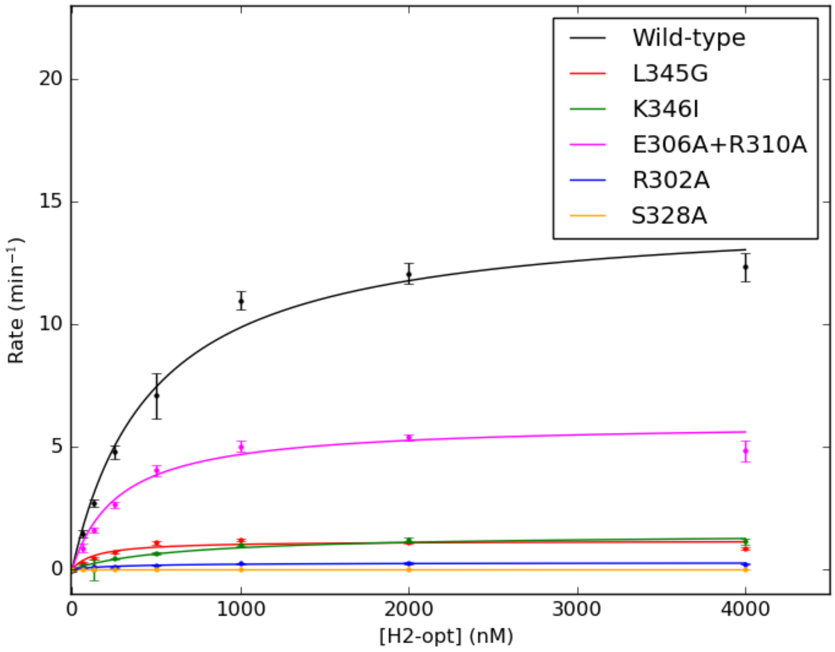

1.9. Figure S2. Implied timescales plot for the 5-state Hidden Markov Model.

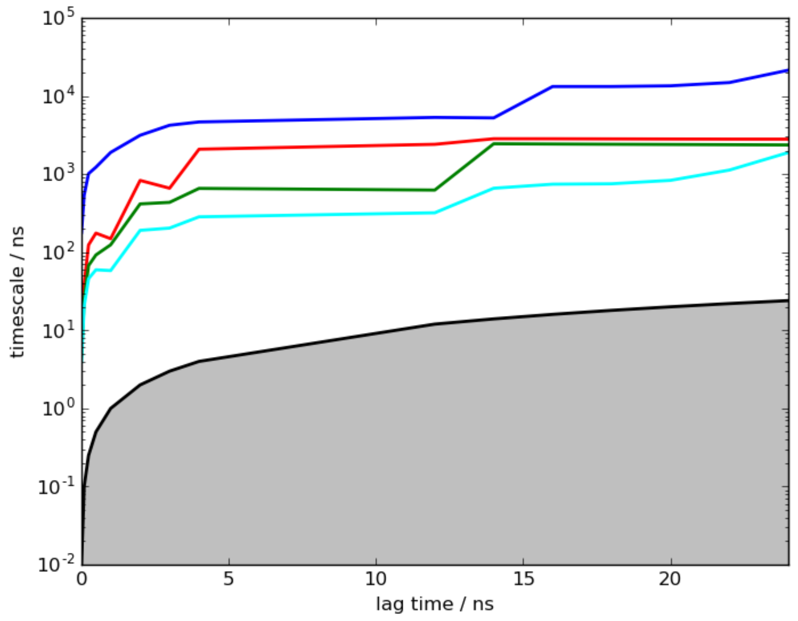

1.10. Figure S3. Dynamic communities of the HtrA1 active, inactive and R302A mutant trimers. Trimer rear view (see front view in main text).

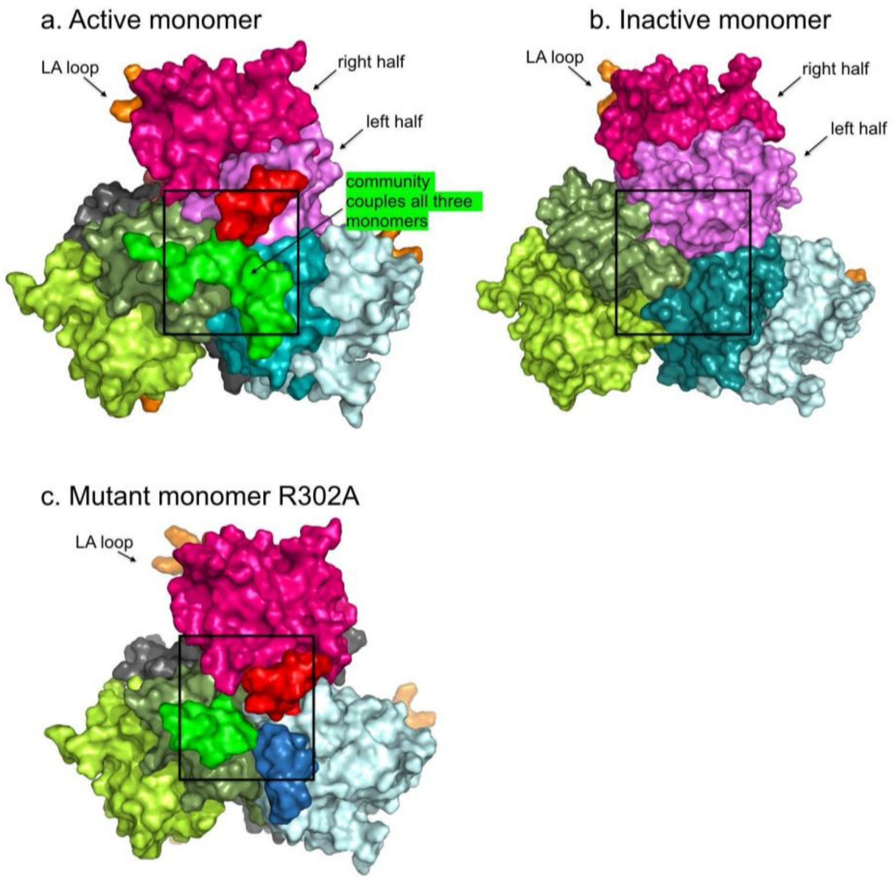

1.11. Supplemental Figure 4: (**a**) WB HtrA1 concentration on bands 54, 47, 37 kD and total HtrA1 (**b**) Total HtrA1 present in cellular samples as measured by intensity in WB. (**c**) raw HtrA1 catalytic activity measured in supernatant from RPE cells.

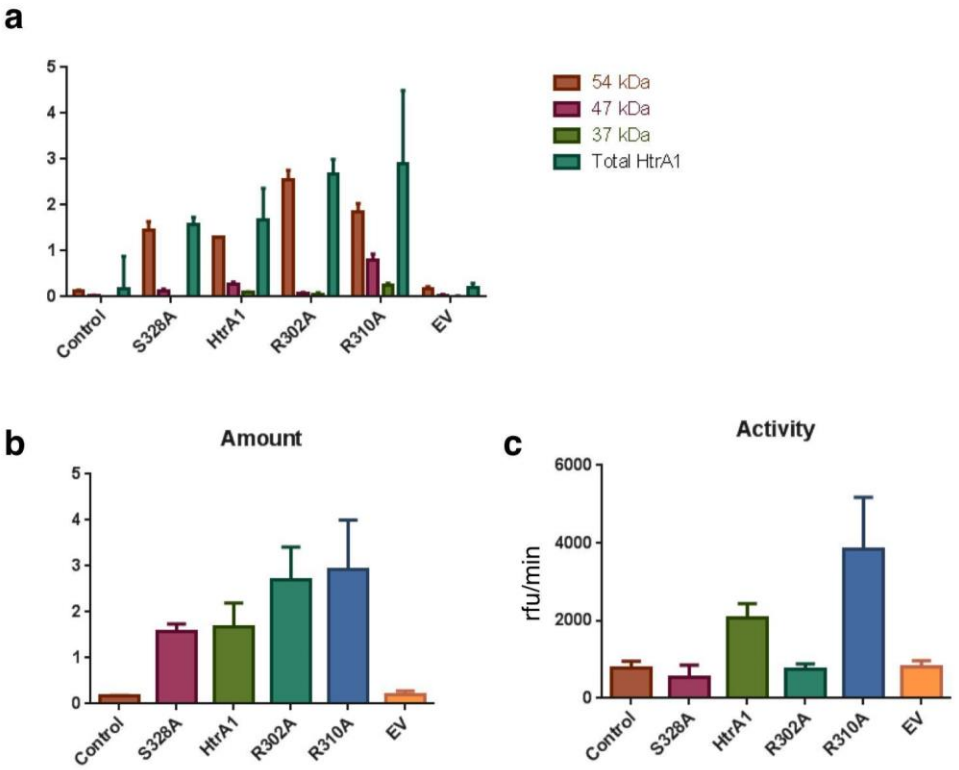

1.12. Proteomics methods

Monitoring HtrA1 activity by targeted proteomics. The putative HtrA1-driven clusterin cleavage site EQL362-NEQ was originally discovered by differential N-terminomic analysis of RPE cell conditioned medium using the Terminal Amine Isotope Labeling of Substrates (TAILS) technique as described elsewhere [13]. In order to validate the presence of the cleaved clusterin peptide in the conditioned media of RPE cells and to confirm its cleavage by HtrA1, a targeted proteomics assay was developed using the mass spectrometry-based selected reaction monitoring (SRM) approach. In addition to the cleaved clusterin peptide, NEQFNWVSR (representing the neo N-terminus) peptides were incorporated into the SRM assay to enable parallel quantification of total HtrA1 and total clusterin. Peptide selection was performed using a combination of experimental and *in silico* approaches, utilising the Skyline software [14], as described in[15]. Isotope-labeled peptides (unpurified), containing either L-[U-^{13}C, U-^{15}N]R or L-[U-^{13}C, U-^{15}N]K, corresponding to the target peptides were synthesized (JPT Peptide Technologies) and their sequences confirmed by LC-MS/MS. SRM method refinement and finalization was then performed using the synthetic peptides in combination with representative biological samples as described in Ref. [15]. (See Supporting Proteomics Data, which describes the SRM parameters used for data acquisition). Conditioned media from RPE cells, which had been transfected with different HtrA1 variants or controls (as described above), was prepared for SRM analysis using an acetone precipitation, on-pellet tryptic digestion protocol. Proteins were precipitated from 100 µl conditioned media by addition of four volumes cold acetone followed by incubation at -20 ºC for 2 h. Samples were then centrifuged at 16,000 g for 10 min at 4 ºC to pellet proteins and supernatants were discarded. The pellets were resuspended in 25 µl 50 mM triethylammonium bicarbonate (TEAB), pH 8.5 containing 0.5 µg proteomics grade trypsin (Roche) by incubation at 37 ºC on a shaker (750 rpm) for 1 h. Cysteines were reduced by addition of 10 µl 35 mM dithiothreitol (DTT), 50 mM TEAB followed by incubation at 60 ºC on a shaker (750 rpm) for 30 min. Cysteine sulfhydryls were then blocked by addition of 10 µl 60 mM iodoacetamide, 50 mM TEAB followed by incubation at 20 ºC in the dark for 30 min. Excess iodoacetamide was quenched by further addition of 10 µl 45 mM DTT, 50 mM TEAB and incubation at 37 ºC on a shaker for 10 min. Finally, 10 µl 50 mM TEAB containing 0.5 µg proteomics grade trypsin (Roche) were added followed by incubation at 37 ºC on a shaker (750 rpm) overnight. Digests were stored at -20 ºC. For SRM analysis, digests (10 µl) were diluted with 10 µl 2% acetonitrile (ACN), 0.1% formic acid (FA) containing: the HtrA1 and clusterin isotope-labelled peptides (~10 fmole/µl); the cleaved clusterin isotope-labelled peptide (~200 fmole/µl); peptides for iRT [16] calibration (8 fmole/µl, Biognosys). SRM analyses were performed on an Ultimate RSLCnano LC coupled to a TSQ Quantiva triple quadrupole mass spectrometer (Thermo Scientific). Samples (5 µL) were loaded at 5 µL/min for 6 min onto a 2 cm × 75 µm C18 trap column (Acclaim Pepmap 100, 3 µm, 300 Å, Thermo Scientific) in loading buffer (0.5% v/v formic acid, 2% v/v ACN). Peptides were then resolved on a 50 cm × 75 µm C18 analytical column with integrated electrospray emitter heated to 40ºC (Easy-SPRAY, 2 µm, 100 Å, Thermo Scientific) using the following gradient at a flow rate of 250 nL/min: 6 min, 98% buffer A (2% ACN, 0.1% formic acid), 2% buffer B (ACN + 0.1% formic acid); 48 min, 30% buffer B; 54 min, 60% buffer B; 56 min, 80% buffer B; 62 min, 80% buffer B; 63 min, 2% buffer B; 86 min, 2% buffer B. The TSQ Quantiva was operated in SRM mode with the following parameters: cycle time, 1.5 s; spray voltage, 1800 V; collision gas pressure, 2 mTorr; Q1 and Q3 resolution, 0.7 FWHM; ion transfer tube temperature 300 ºC.

The MS data were processed in Skyline version 3.5.0.9319[14]. The quality control and normalization were performed using the MSstats package version 3.6.0 (www.msstats.org)[17] in R version 3.3.2 (www.r-project.org)[18]. Peptide intensity values were $\log_2$ transformed and the median intensities were equalized across all heavy-labeled reference peptides. Peptide summarization to protein abundance was performed using Tukey's median polish, and the cleaved clusterin peptide NEQFNWVSR was treated as a separate protein. Clusterin cleavage was assessed by adjusting the cleaved peptide abundance for HtrA1 and clusterin content present in the samples, acquired as the residuals of a model containing the two protein amounts. Statistical modeling was performed on these adjusted cleavage values, taking into account the experimental factors and the six different cell types. The following comparisons were made, adjusted for multiple testing [12]: HtrA1 − EV, S328A − EV, HtrA1 − S328A, R302A − HtrA1, R310A − HtrA1, and R302A − S328A. More details can be found in Supporting Statistics.

Figure S5: (Top) Representative chromatograms for the cleaved clusterin peptide NEQFNWVSR and the corresponding stable isotope-labelled internal standard. Specificity is determined by co-elution of the cleaved clusterin peptide with the internal standard in addition to the matching transition ratios. (Bottom) Peptide abundances without adjustment for clusterin (CLUS) and HTRA1 protein level. (More information in Supplemental Statistics.html)

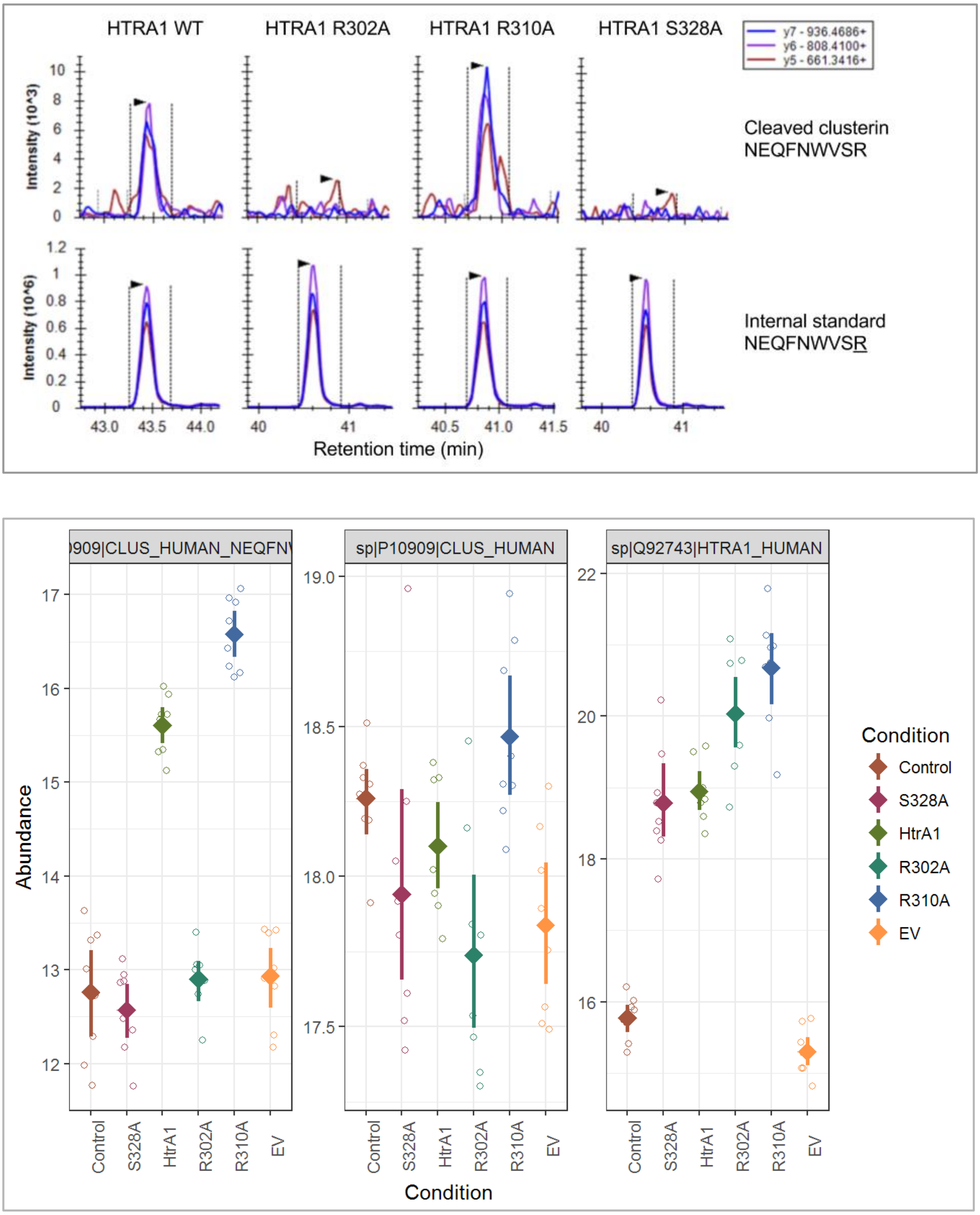

1.13. Supplemental native mass-spectrometry data.

In all graphical representations, the upper trace (C) contains the annotation of the peaks. On top the mass/charge (m/z) ratio for the species, the indicated m/z value with its intensities below and a graphical indicator. This indicator marks species (different colors for different species) in the sample with a single dot for a monomer or an assembly of three dots for a trimer).

HtrA1 wild-type catalytic domain (Panel 1). The protein solution contains an unmodified (green; A) and possibly N-glycosylated species (red). Therefore, the trimeric species are mixed between these species in different ratios resulting in very broad trimeric peaks. At high backing pressure (low-trace, panel A) mostly trimer was observed (ratio trimer/monomer = 6/2 → 3) whereas increasing collision energy and/or lowering backing pressure results in an increase in monomeric species and a shift to higher m/z values for the trimer indicating a slightly loss of the native folded trimeric state.

HtrA1 mutant R302A (Panel 2)**.** The protein (green) is in high trimeric state at initial conditions. At high backing pressure (low trace, panel A) mostly trimer was observed (ratio trimer/monomer = 8/3 → ~2.7) whereas increasing collision energy and/or lowering backing pressure results in an increase in monomeric species similar to the HtrA1 wild-type. Also, in this sample, higher collision energy or backing pressure increase m/z ratio indicating a slightly loss of native folded trimeric state. The lower trace (A) indicating high trimer formation with some sodium and ammonium adducts present. At higher collision energy (B) and/or lower backing pressure (C) mass resolution increased for the trimer (smaller peaks) but also monomer generated. Also m/z ratios shifted to higher values indicating slightly loss of native conformation.

HtrA1 mutant S328A (Panel 3). The protein solution contains an unmodified (green), an N-glycosylated species (red) and another not specified species (blue). Therefore, the trimeric species are heavily mixed between these species which results in very broad trimeric peaks (for green & red trimers) and an additional trimer consists of one blue species and one or two red (or green) monomers. At higher backing pressure (low trace) trimer to monomer ratios of 4 (22/5 → ~4) was observed whereas at increasing collision energy and/or lower backing pressure the ratio increased to ~1.4 towards monomeric species. Also, a shift to higher m/z values for the trimer indicating a slightly loss of the native folded trimeric state.

1.14. Figure S7. Results for L3 loop conformation analysis. A) Active conformation (PDB 3NZI); B) An inactive conformation model.

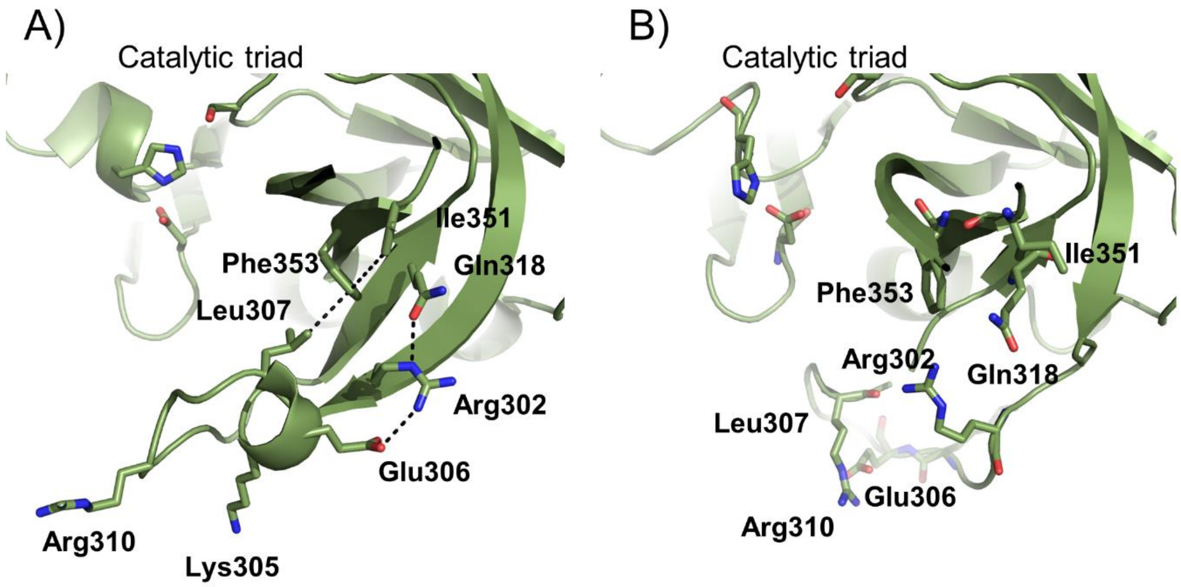

1.15. Supplemental Community Analysis HtrA1 monomer

Using computational community analysis, a total number of 8 and 9 dynamic communities were found in the active state and inactive states respectively (Supplemental Figure 7 a, b and Supplemental Table 5). In both instances (i.e. active, inactive monomer), a few dynamic communities remain common. Community 3 comprises the catalytic triad, as well as the core of the protein. Interestingly, it also includes residues in the LC loop (i.e. Lys248, Lys243 and Thr223) which have been recently reported in connection with the allosteric mechanism of inhibition of an anti-HtrA1 antibody by Ciferri et al. [1]. The authors of that work proposed an allosteric inhibition of HtrA1 by the antibody bound far from the catalytic site, which is supported by our community model that shows that residues in the LC loop are coupled with the motions of the catalytic triad. In this context, by interacting with the LC loop, the antibody could be perturbing the dynamics of this amino acid community, and therefore reduce the proteolytic activity remotely, without directly interacting with the catalytic triad, as in other antibody-protein complexes of the same family. Community 7, on the other hand, consists of both the oxyanion hole forming loop and the loop L2 which according to our MD simulations cooperate in the activation process (Section 1). In our model, the oxyanion hole stabilizes the transition state of the reaction and the loop L2, interacts with the substrate in the active state, and blocks the active site as a lid in the inactive state. Finally, community 2 contains the LA loop, which has been identified as an important regulatory element in bacterial homologs but whose function is not well-understood in the HtrA1 protein.

The main differences between the inactive and active monomer states reside in the cooperativity between the LD loop (residue Ser284 to Asn290), the oxyanion hole forming loop and the L2 loop elements (Supplemental Figure 8). In the inactive state, the LD loop is coupled with the blocked peptide-binding site and the L2 loop is found in community with the L3 loop (Community 6). In contrast, in the active state, the LD loop seems to act as an independent block (Community 5b), and the L2 loop is dynamically coupled with the oxyanion hole and the peptide binding site (Community 7). This dynamic pattern points to an interplay between the L2, L3 and LD loops in combination with the enzyme active site. Importantly, the LD loop is found in the trimeric structure directly interacting with the L3 loop of the contiguous monomer, pointing to the possibility of a signal transduction across monomer interfaces via the LD-L3 system. We conclude that global picture that includes the whole trimer would be needed for understanding HtrA1 dynamics, which we address in the main manuscript.

1.16. Supplemental analysis of the L3 loop conformations

The changes that occur in the HtrA1 L3 loop throughout the activation process can be considered an order-disordered transition, where the disordered loop in the inactive and intermediate conformations evolves to a more ordered conformation that can be observed in the active form by X-ray crystallography (PDB ID 3NZI). In order to investigate this transition, we performed an analysis of the segment between residues 298 and 310 using the combination of our molecular dynamics simulations and markovian models applied to the full protein, the HtrA1 and DegS X-ray structures available and the extensive mutagenesis works of de Regt et al.[2,3] on other bacterial HtrA proteases (DegS and DegP).

We found three groups of residues to be strongly involved in the process (Figure S7): a) Arg302, Glu306, Gln318; b) Leu307, Phe353 and Ile351; and c) Lys305 and Arg310.

a) The first group contains the residue Arg302 which has been found in this work to be essential to transmit the signal to other monomers in the trimer. Residues Glu306 and Gln318 interact with Arg302, essentially locking its position in the active conformation. Gln318, specially, occupies the Arg302 position in the inactive conformation and it is therefore displaced by Arg302 when the activation process occurs. De Regt et al.[3] have found that Gln191, the equivalent residue to Gln318 (HtrA1) in the bacterial HtrA protease DegS, seems to be essential and its mutation to alanine abolish the enzymatic activity, suggesting that it might play an equivalent role in HtrA1. Regarding Glu306, the double mutant E306A+R310A showed an important decrease in activity, while the R310A mutant was unaffected, pointing to a deleterious effect of E306A for the enzymatic activity in agreement with our model.

b) Leu307, Phe353 and Ile351 form a network of hydrophobic contacts that are related to the conformations of Gln318 and the L2 loop. Leu307 (L3 loop) interact with Phe353 only in the active conformations, while it is not possible in the inactive state of the protein due to the presence of the Lys346, that occupies the same spot. Phe353 interacts in turn with Ile351, whose position depends on the conformation of the L2 loop. In the inactive state, the L2 loop adopts a twist (due to the flip of the Leu345 and Lys346 side chains) that forces Ile351 into pushing Gln318 in the Arg302 active spot, preventing activation. Only when Lys346 flips back, Ile351 can adopt a favourable conformation to interact with Phe353 which in turn interacts with Leu307. The essential role of this network of interactions is highlighted once more by its conservation in other members of the family (DegS) where mutations abolish completely the enzymatic activity[3].

c) Lys305 and Arg310. These two residues appear to work facilitating the conformational change by producing a high degree of flexibility in the L3 loop. The role of Arg310 has been evaluated by expressing the full protein R310A variant in the RPE cells where it presents a slightly higher autocleavage and general enzymatic activity according to our western blot and biochemical assay respectively, indicating that the removal of the loop flexibility, to a certain extent, may favour the ordered conformation of the L3 loop.

Figure S6: (Panel 1) HtrA1 wild-type catalytic domain, (Panel 2) mutant R302A (Panel 3) S328A. (A) lower trace (B) higher collision energy, (C) lower backing pressure

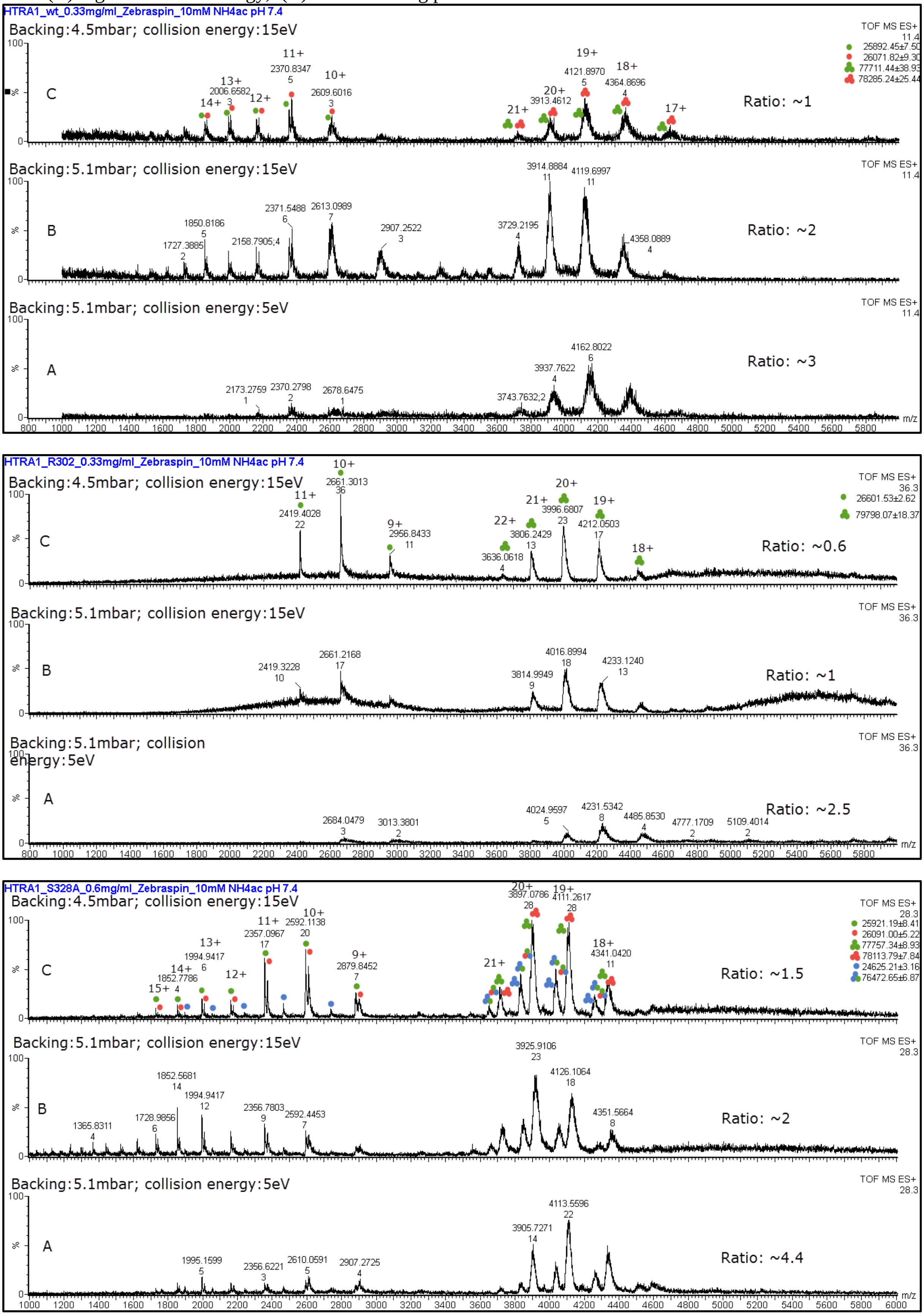

Figure S7: Dynamical communities of the HtrA1 monomer. Highlighted in bold, communities that differ between active and inactive states. Ribbons correspond to the contiguous monomer (excluded of this analysis), as reference. (a) Active form. Total of 8 dynamic communities. Community 6b (LD loop) does not exist in the inactive monomer. The L2 loop is part of Community 7, coupled to the oxyanion hole. (b) Inactive form. Community 3 is unique to the inactive monomer. The L2 loop is coupled to the L3 loop (Community 6). The LD loop is part of Community 7.

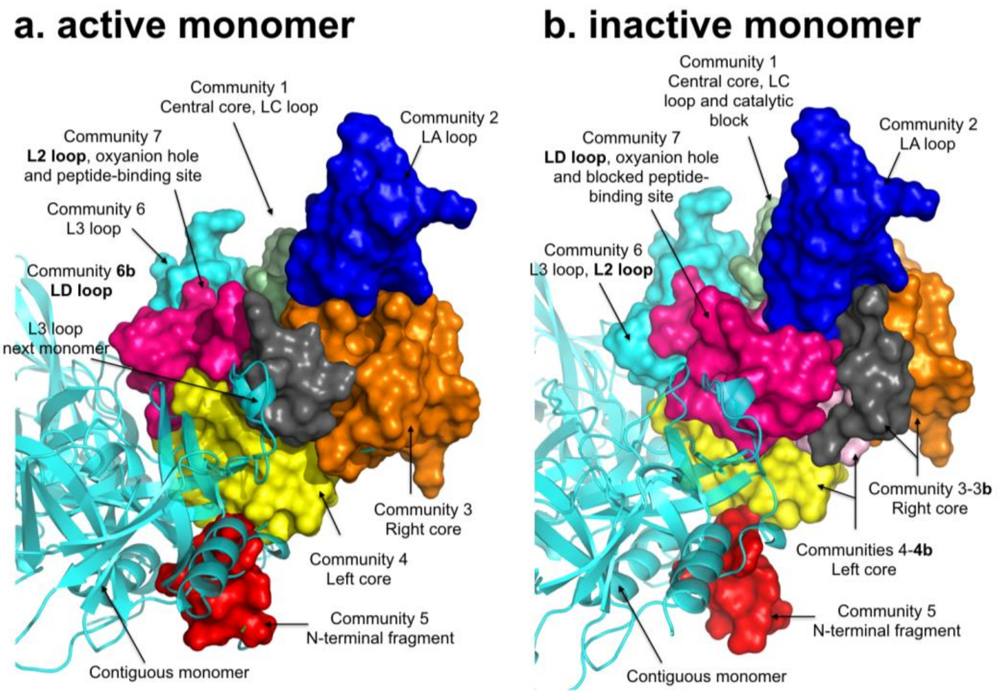

2. Supplemental Experimental Procedures

2.1. Simulation and modelling details

2.1.1. Simulation. Proteins were solvated in a TIP3P waters box with a minimum distance to the edge of 12 Å. All structures were energetically minimized using the steepest descent algorithm to a maximum force of 100 kJ mol^{-1} nm^{-1}, then, 100 ps of NVT molecular dynamics simulation at 310K with non-hydrogen atoms restraint with a force of 1000 kJ mol^{-1} nm^{-2} were carried out, followed by another 100 ps of NPT simulation with restraints (310K, 1bar). Finally, unrestrained production simulations of lengths between 50 ns and 1000 ns (Supplemental Table 1), saving the Cartesian coordinates each 10 ps, were obtained to produce a total of 14.23 µs of simulation time. Each trajectory was split in three different monomeric trajectories and aggregated to yield a total of 42.69 µs for analysis. A cut-off of 10 Å was used for van der Waals and electrostatic short-range interaction while PME was used for long-range interactions. The V-rescale thermostat and the Parinella-Rahman barostat were employed to maintain temperature and pressure respectively in the simulations.

2.1.2. Community analysis. To describe the dynamic units of HtrA1 we have used the *MutInf* method described by McClendon et al.[4], which uses mutual information to identify correlated movements based on simulations in the nanoseconds scale. Phi, psi, chi1 and chi2 dihedral angles were used with a discretization of 15 degrees, and the first 10 ns of each simulation were discarded to account for equilibration of the simulations. For the subsequent community analysis, a minimum mutual information threshold of 0.05 was established together with a minimum contact simulation time between residues of 75%. Residue contact cut-offs were studied ranging from 7 to 12 Å. Finally, as described by McClendon et al.[5], we have employed the Girvan-Newman algorithm[6] to detect the dynamic communities in our monomeric and trimeric systems. Distance cut-offs of 5 Å and 7 Å provided optimal resolution regarding the size and number of communities in the monomer and trimer studies, respectively (Supplemental Tables 2 and 3).

2.1.3. Helicity analysis. The DSSP program was used to assign the secondary structure to the residues in LD loop (P285 to S287) for each simulation snapshot. Wild-type and variant monomers aggregated simulations, starting from an active conformation, were used by getting snapshots each 5 ns. The percentage of simulation time assigned to a helix was measured by residue.

2.1.4. Hidden Markov model's construction. Using the PyEMMA package[7], and MDTraj[8], we replaced the Cartesian coordinates of our circa 2.1 million snapshots from the molecular dynamic simulations for the phi, psi and chi dihedral angles of the protein residues. Then, we applied the time-lagged independent component analysis (TICA) to reduce the coordinates using the two first components. Following, we used the k-means algorithm to cluster the conformations into 1472 microstates, based on the general rule of thumb for the number of clusters that should be equal to the squared root of the number of samples described by Scherer et al. The clustered trajectories were used to build Hidden Markov Models with 3 to 9 states and lag times of 10 ps, 0.1 ns, 1 ns to 10 ns in 1 ns intervals and from 10 ns to 20 ns in 2 ns intervals. Implied timescales plots and results for the Chapman-Kolmogorov test were generated for each combination. A model with 5 hidden states and a lag time of 5 ns was chosen and the Transition Path Theory framework[9] was used to identify the pathway between the inactive and active states with the highest reactive flux.

2.1.5. Residue conservation analysis. Sequences from 120 HtrA-family related proteins were downloaded from Uniprot[10] and aligned using kalign[11]. Frequency of the different amino acid types were calculated for HtrA1 positions Leu345 and Lys346.

2.2. Statistics for Proteomics

Peptide intensity values were log2 transformed and the median intensities were equalized across all heavy-labelled reference peptides. Peptide summarization to protein abundance was performed using Tukey's median polish, and the cleaved clusterin peptide NEQFNWVSR was treated as a separate protein. Clusterin cleavage was assessed by adjusting the cleaved peptide abundance for HtrA1 and clusterin content present in the samples, acquired as the residuals of a model containing the two protein amounts. Statistical modelling was performed on these adjusted cleavage values, considering the experimental factors and the six different cell types. The following comparisons were made, adjusted for multiple testing[12]: HtrA1 − EV, S328A − EV, HtrA1 − S328A, R302A − HtrA1, R310A − HtrA1, and R302A − S328A. More details can be found in Supplemental Statistics (accessory .html file).

2.3. Western Blot analysis.

Cells were washed with PBS and lysed with RIPA buffer (Thermo Scientific) containing protease inhibitors (Roche Diagnostics GmbH, Mannheim, Germany). 200µl cell media was precipitated with ice cold acetone at least 1h at -20°C and then centrifuged at 16,000g for 10 min. Cell pellet proteins were then dissolved with RIPA buffer containing anti-protease. Samples (25µg per cells, 20 µl per media) were then denatured in NuPage® LDS Sample buffer 4X (Invitrogen, UK) at 70°C for 10min and run on commercially produced pre-cast 4–15% Criterion TGX strain-free gels (Bio-Rad) with Tris/Glycine/SDS (TGS) buffer (Bio-Rad). The proteins were transferred to a Trans-Blot® Turbo™ (Bio-Rad) membrane using the Trans-Blot® Turbo™ Transfer System (Bio-Rad) for 7 minutes. Membranes were incubated with 5% Blotting Grade Blocker non-fat Dry Milk (Bio-Rad) in Tris-buffered saline (TBS) (Sigma) + 0.05% Tween-20 (Sigma) for 1h at RT prior to incubation with primary antibody specific against HtrA1 (1:1000, (Vierkotten et al., 2011)), and GAPDH (64920, Biolegend) as the loading control, overnight at 4°C. Immunodetection was performed by incubating the membranes with secondary antibodies IRDye® 680CW donkey anti-rabbit IgG (H + L), and IRDye® 800CW donkey anti-mouse IgG (H + L), (1:5000, LI-COR Bioscience, Lincoln, NE) for 1h at RT prior to washing with TBS + 0.05% Tween-20. Immunoreactive bands were visualized using the LI-COR Odyssey® infrared imaging system (LI-COR Bioscience, Lincoln, NE) according to the manufacturer's specifications.

Figure S8. Original Western Blot Image. Figure 4 in the main text was cropped from the left blot.

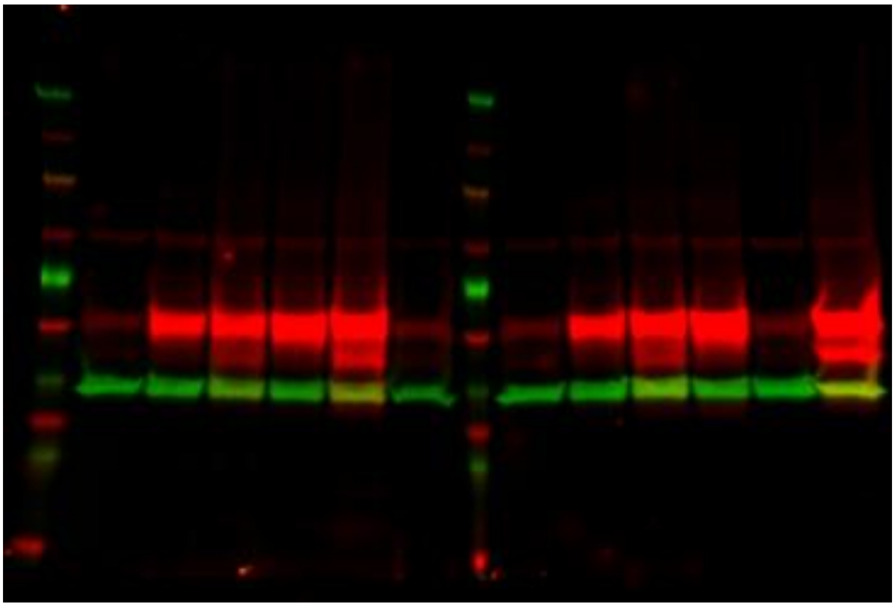

3. Supplemental References

1 Ciferri, C. *et al.* The trimeric serine protease HtrA1 forms a cage-like inhibition complex with an anti-HtrA1 antibody. *Biochemical Journal* **472**, 169-181 (2015).

2 De Regt, A. K., Baker, T. A. & Sauer, R. T. Steric clashes with bound OMP peptides activate the DegS stress-response protease. *Proceedings of the National Academy of Sciences* **112**, 3326-3331 (2015).

3 De Regt, A. K. *et al.* A conserved activation cluster is required for allosteric communication in HtrA-family proteases. *Structure* **23**, 517-526 (2015).

4 McClendon, C. L., Friedland, G., Mobley, D. L., Amirkhani, H. & Jacobson, M. P. Quantifying correlations between allosteric sites in thermodynamic ensembles. *Journal of chemical theory and computation* **5**, 2486-2502 (2009).

5 McClendon, C. L., Kornev, A. P., Gilson, M. K. & Taylor, S. S. Dynamic architecture of a protein kinase. *Proc Natl Acad Sci U S A* **111**, E4623-4631, doi:10.1073/pnas.1418402111 (2014).

6 Girvan, M. & Newman, M. E. Community structure in social and biological networks. *Proceedings of the national academy of sciences* **99**, 7821-7826 (2002).

7 Scherer, M. K. *et al.* PyEMMA 2: A software package for estimation, validation, and analysis of Markov models. *Journal of chemical theory and computation* **11**, 5525-5542 (2015).

8 McGibbon, R. T. *et al.* MDTraj: A modern open library for the analysis of molecular dynamics trajectories. *Biophysical journal* **109**, 1528-1532 (2015).

9 Vanden-Eijnden, E. Transition-path theory and path-finding algorithms for the study of rare events. *Annual review of physical chemistry* **61**, 391-420 (2010).

10 Consortium, U. UniProt: a hub for protein information. *Nucleic acids research*, gku989 (2014).

11 Lassmann, T. & Sonnhammer, E. L. Kalign–an accurate and fast multiple sequence alignment algorithm. *BMC bioinformatics* **6**, 1 (2005).

12 Hothorn, T., Bretz, F. & Westfall, P. Simultaneous inference in general parametric models. *Biometrical journal* **50**, 346-363 (2008).

13 Lai ZW, Petrera A, Schilling O. Protein amino-terminal modifications and proteomic approaches for N-terminal profiling. Current opinion in chemical biology 24, 71-79 (2015).

14 MacLean B, et al. Skyline: an open source document editor for creating and analyzing targeted proteomics experiments. Bioinformatics 26, 966-968 (2010).

15 Dunkley T, et al. Characterization of a human pluripotent stem cell-derived model of neuronal development using multiplexed targeted proteomics. PROTEOMICS-Clinical Applications 9, 684-694 (2015).

16 Escher C, et al. Using iRT, a normalized retention time for more targeted measurement of peptides. Proteomics 12, 1111-1121 (2012).

17 Choi M, et al. MSstats: an R package for statistical analysis of quantitative mass spectrometry-based proteomic experiments. Bioinformatics, btu305 (2014).

18 R Core Team (2013). R: A language and environment for statistical computing. R Foundation for Statistical Computing, Vienna, Austria. URL http://www.R-project.org/
